# Mutation-Independent Therapies for Retinal Diseases: Focus on Gene-Based Approaches

**DOI:** 10.3389/fnins.2020.588234

**Published:** 2020-09-24

**Authors:** Sabrina Carrella, Alessia Indrieri, Brunella Franco, Sandro Banfi

**Affiliations:** ^1^Telethon Institute of Genetics and Medicine (TIGEM), Pozzuoli, Italy; ^2^Medical Genetics, Department of Precision Medicine, University of Campania “Luigi Vanvitelli”, Naples, Italy; ^3^Institute for Genetic and Biomedical Research (IRGB), National Research Council (CNR), Milan, Italy; ^4^Medical Genetics, Department of Translational Medical Sciences, University of Naples Federico II, Naples, Italy

**Keywords:** retinal disease, mutation-independent, gene therapy, neuroprotection, microRNA, genome editing, optogenetics

## Abstract

Gene therapy is proving to be an effective approach to treat or prevent ocular diseases ensuring a targeted, stable, and regulated introduction of exogenous genetic material with therapeutic action. Retinal diseases can be broadly categorized into two groups, namely monogenic and complex (multifactorial) forms. The high genetic heterogeneity of monogenic forms represents a significant limitation to the application of gene-specific therapeutic strategies for a significant fraction of patients. Therefore, mutation-independent therapeutic strategies, acting on common pathways that underly retinal damage, are gaining interest as complementary/alternative approaches for retinal diseases. This review will provide an overview of mutation-independent strategies that rely on the modulation in the retina of key genes regulating such crucial degenerative pathways. In particular, we will describe how gene-based approaches explore the use of neurotrophic factors, microRNAs (miRNAs), genome editing and optogenetics in order to restore/prolong visual function in both outer and inner retinal diseases. We predict that the exploitation of gene delivery procedures applied to mutation/gene independent approaches may provide the answer to the unmet therapeutic need of a large fraction of patients with genetically heterogeneous and complex retinal diseases.

## Introduction

**FIGURE 1:**
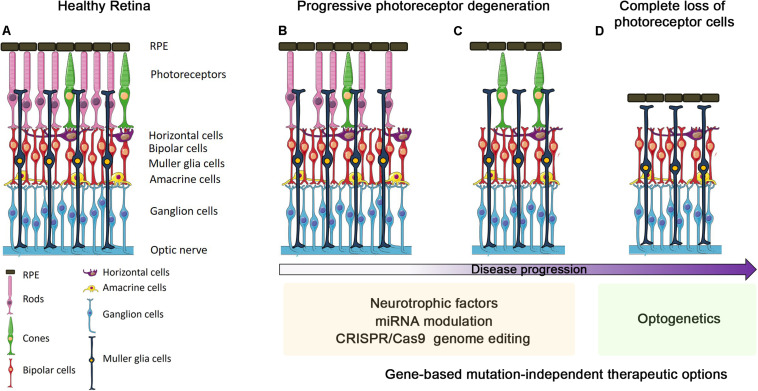
Progression of outer retinal degenerative disease and gene-based mutation-independent therapy options. Schematic representation of either retinas in healthy conditions **(A)** or undergoing different stages of degeneration progression **(B–D)**. The ongoing photoreceptor degeneration **(B)** and before the progressive cone degeneration **(C)** stages can benefit from the use of neurotrophic factors, miRNA modulation and CRISPR/Cas9 genome editing. In the final stage, i.e., when all photoreceptors are lost **(D)**, optogenetics seems to be the only suitable gene-based mutation-independent therapy option.

The retina is the neuronal tissue in charge of visual function. It comprises five main neuronal types that form morphologically and functionally distinct circuits working, in parallel and in combination, to produce a complex visual output (Hoon et al., 2014; Figure 1A). The outer retina (OR) is composed of the Retinal Pigment Epithelium (RPE) and of photoreceptors. The RPE is a monolayer of pigmented cells that contribute to the visual cycle and provide metabolic support to photoreceptors. The latter, subdivided into rods and cones, are the neurons in charge to convert light energy into membrane potential changes in the phototransduction cascade. Rod and cone photoreceptors synapse with neurons of the inner retina (IR), namely bipolar cells, which in turn contact retinal ganglion cells (RGCs) and amacrine cells. The sole output neurons of the retina are RGCs, which project axons, forming the optic nerve, to higher visual centers in the brain (Hoon et al., 2014). Besides neurons, the retina contains a glial component, represented by Müller cells, astrocytes, and the microglia. Müller cells account for about 90% of the retinal glia and, together with astrocytes, provide neurotrophic, metabolic and mechanical support to neurons. Microglia constitute the resident immune cell population in the retina and orchestrate neuroinflammatory response, recovery from injury and progression of disease (Rashid et al., 2019).

Most retinal diseases can be categorized into those involving death of photoreceptors in the OR, and those directly affecting neurons of the IR, usually RGCs. Retinal diseases affecting the OR are the most important causes of vision impairment in the working-age population and include: (a) inherited disorders, e.g., retinitis pigmentosa (RP), Leber congenital amaurosis, and macular dystrophies, which display high genetic heterogeneity with more than 250 causative genes^1^ and (b) multifactorial conditions, e.g., AMD. Degeneration of RPE and/or photoreceptor cells are the common landmarks of these diseases, although the underlying molecular and cellular events are still poorly understood. Mitochondrial dysfunction, neuroinflammation and microglia activation, among others, have been shown to exacerbate disease progression (Ambati et al., 2013; Cuenca et al., 2014; Zhao et al., 2015; Lefevere et al., 2017). Schematically, the disease proceeds through three different stages (Figures 1B–D): (a) functional impairment, in which photoreceptors are intact but start to lose their functionality; (b) ongoing photoreceptors degeneration; (c) complete loss of photoreceptors. The choice of the appropriate therapy may depend on the specific timing of intervention (Figures 1B–D).

**FIGURE 2:**
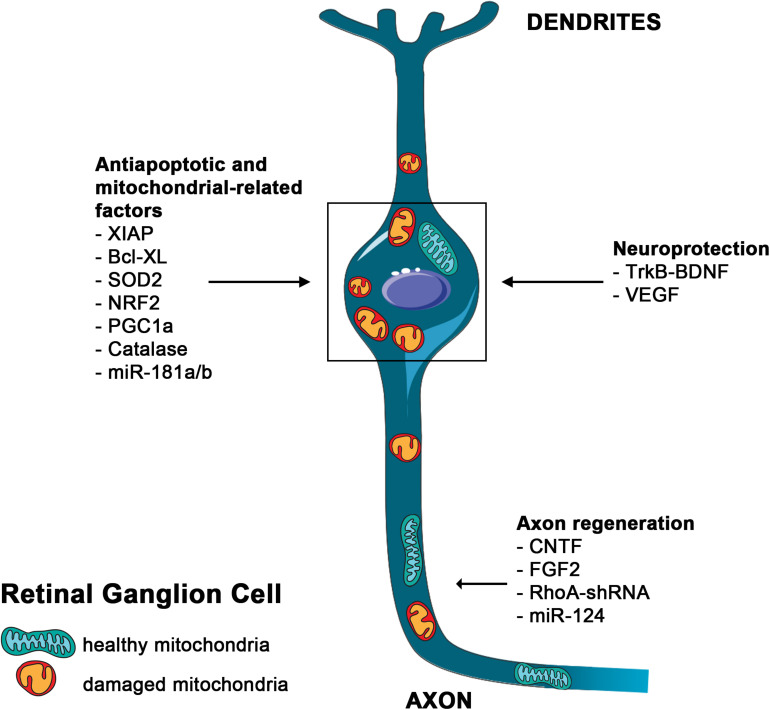
Main mutation-independent therapies applicable to RGC neuroprotection. Schematic representation of an RGC. Damaged mitochondria are depicted in the soma and along the axon. Therapies aimed to block RGCs death and to induce axon regeneration are indicated.

Inner retina diseases are mostly represented by optic neuropathies (ONs), that comprise a group of monogenic and complex disorders mainly affecting RGCs (Carelli et al., 2017). The mechanisms underlying RGC degeneration are still unknown, but axonal injury has been proposed as the earliest event that eventually leads to RGC death (Dratviman-Storobinsky et al., 2008). Among the possible mechanisms, mitochondrial dysfunction, has been correlated, directly or indirectly, to the majority of ONs, likely due to the particular vulnerability of RGCs to mitochondrial impairment (Carelli et al., 2017). A tight connection between ON and mitochondrial dysfunction has been extensively described (Gueven et al., 2017). Several mitochondrial diseases are associated with vision impairment. Among them, Leber Hereditary Optic Neuropathy (LHON) and Autosomal Dominant Optic Atrophy represent the most frequent genetic form of ONs (Carelli et al., 2004; Yu-Wai-Man et al., 2011). Interestingly, also the most common and multifactorial forms of ONs, such as glaucoma and diabetic retinopathy, show signs of mitochondrial dysfunction and share clinical similarities with mitochondrial ONs (Jarrett et al., 2008; Yu-Wai-Man et al., 2011; Chhetri and Gueven, 2016; Gueven et al., 2017). Despite the efforts employed, ONs are still only symptomatically treated. In this respect, mitochondria could represent the common denominator and hence a promising common therapeutic target (Figure 2).

### Mutation-Independent Strategies Based on Gene-Therapy Approaches

Gene-based therapy consists in the delivery of exogenous genetic material in order to modulate the expression of specific genes to treat pathological conditions. This approach ensures a targeted, stable and spatially regulated modulation of gene expression, either through overexpression or silencing, with a final therapeutic outcome and was initially devised for gene-specific applications. Adeno-associated viruses (AAV) represent the vectors of choice for gene therapy in the retina, due to the limitation of lentiviruses and adenoviruses in transducing the mature retina. AAVs have an excellent safety profile (lack of pathogenicity and low immunogenicity) and provide long-lasting transgene expression (Colella and Auricchio, 2010; Trapani, 2019). Diseases caused by loss-of-function mutations can be treated by gene-replacement therapy. Indeed, the success of clinical trials and the recent approval of Luxturna (Ledford, 2017; Apte, 2018) are laying the bases for a more widespread use of gene replacement strategies for the treatment of retinal diseases. However, the extensive genetic heterogeneity of both OR and IR diseases poses a significant limitation to the development and application of gene-specific strategies to the majority of patients. Another problem is that AAV particles can package only up to about 4.7 kilobases of DNA, which represent a serious obstacle to the delivery of larger genes (Trapani, 2018, 2019). Moreover, gene replacement is not effective, by itself, in diseases caused by gain-of-function mutations, which primarily require silencing of mutant alleles. Finally, gene replacement therapies at present cannot be applied to complex multifactorial retinal diseases, including age-related macular degeneration (AMD), glaucoma, and diabetic retinopathy which are characterized by a polygenic, not fully elucidated, pathogenicity, with the contribution of environmental factors.

For the above-mentioned reasons, mutation-independent therapeutic strategies, represent valid alternatives and/or complementary approaches to gene-replacement strategies. They aim at minimizing and/or delaying cell loss in the diseased retina regardless of the primary genetic defect, targeting common dysregulated pathways that play a key effector role in retinal damage (e.g., oxidative stress, inflammation, neovascularization, pyroptosis, etc.).

It is becoming clear that gene-based strategies can considerably enhance the efficacy and safety of mutation-independent therapies for retinal diseases that, to date, are mainly based on the use of therapeutic active compounds. Indeed, their systemic administration may not be always efficient considering that many molecules cannot pass the blood–retinal barrier (Almasieh and Levin, 2017). Moreover, compounds intraocular administration can be deleterious because requires repeated local injections that may cause endophthalmitis and retinal detachment. In this respect, the exploitation of viral vector-mediated intraocular delivery is raising considerable interest for the devise of mutation-independent therapies because it ensures a more stable expression and efficacy of the therapeutic agent and usually requires a single administration, safeguarding patients’ welfare. Finally, the appropriate combination of specific vector serotypes and cell type−specific promoters would limit transgene expression to the desired cells.

In this review, we will discuss the technical and conceptual reasons underlying the advantages of AAV delivery- and more in general, of gene-based therapies applied to mutation-independent strategies for the treatment of OR and IR diseases and the most promising applications reported to date.

### Neuroprotection Approaches

Neuroprotective strategies are the ideal mutation-independent approach to treat diseases with initial and/or ongoing photoreceptor degeneration (Figures 1B,C). However, the half-life of neurotrophic factors is in general short thus requiring frequent administrations to maintain appropriate therapeutic levels (Poduslo and Curran, 1996). On the contrary, AAV-mediated expression of neurotrophic factors can ensure stable transgene expression and therapeutic efficacy. Glial cell–derived neurotrophic factor, ciliary neurotrophic factor (CNTF), brain-derived neurotrophic factor (BDNF), basic fibroblast growth factor, and pigment epithelium–derived factor (PEDF) were shown to have beneficial effects in preventing photoreceptor death in several mouse models of retinal degeneration (Unsicker, 2013; Paulus and Campbell, 2016; Pardue and Allen, 2018). Numerous studies have suggested that CNTF and the activation of IL-6 signaling, also through overexpression of STAT3 (Jiang et al., 2014), are important in the response to retinal degeneration although treatment with CNTF has shown limited efficacy in RP patients (Birch et al., 2016).

Neurotrophic agents have also been tested in ONs (Boia et al., 2020; Figure 2). AAV-mediated delivery of Fibroblast growth factor-2, or delivery of the *BDNF* gene leads to significant increase in RGC survival and reduced RGC axons loss after optic nerve injury or induction of intraocular pressure (IOP) (Martin et al., 2003; Sapieha et al., 2006).

More recently, a single AAV vector expressing both BDNF and its receptor, the tropomyosin-related receptor kinase-B (TrkB), was tested *in vivo* in a model of optic nerve crush and in a model of high tension glaucoma (HTG) obtained by trabecular laser-induced IOP elevation (Osborne et al., 2018). This strategy promoted significant long-term RGCs survival and improved positive scotopic threshold responses (measuring RGCs activity) after optic nerve crush or laser-induced IOP elevation (Osborne et al., 2018).

Similarly, the effect of an AAV-mediated CNTF gene therapy was tested in different models of RGC degeneration, such as optic nerve crush and laser-induced HTG with promising results (Leaver et al., 2006; Pease et al., 2009). Interestingly, co-administration of CNTF and BDNF vectors did not improve RGC survival, possibly because this combination reduced the efficiency of AAV-CNTF compared to a single vector administration (Pease et al., 2009).

Conversely, combined overexpression of CNTF and Bcl-2, or CNTF and RhoA-shRNA were tested in models of optic nerve injury (Leaver et al., 2006; Cen et al., 2017). Inhibition of the Rho GTPase signaling pathway by RhoA downregulation led to enhancement of axon regrowth (Fujita and Yamashita, 2014). Interestingly, both strategies demonstrated additive effects on increased survival and axonal regeneration of RGCs highlighting the importance of a therapeutic approach enhancing simultaneously neuroprotection and regeneration in ON (Leaver et al., 2006; Cen et al., 2017).

Within neuroprotection, a special place is occupied by the rod-derived cone viability factor (RdCVF) whose discovery and characterization uncovered an entirely novel mechanism of neuroprotection. Most forms of RP are due to primary damage of rod cells, which is followed by a secondary loss of cones (Figure 1C; Sancho-Pelluz et al., 2008). RdCVF is a retina-specific trophic factor that induces cone survival and functional rescue in RP animal models (Leveillard et al., 2004; Yang et al., 2009) by modulating energy metabolism, accelerating glucose entry and enhancing aerobic glycolysis, thus holding good promises as an effective AAV-mediated mutation-independent therapy (Leveillard and Sahel, 2010; Ait-Ali et al., 2015; Byrne et al., 2015; Clerin et al., 2020).

Neurotrophic factors have also been tested in combination with other mutation-independent therapeutic agents. PEDF displays antiangiogenic and anti-inflammatory properties and was tested in combination with the microRNA-mediated inhibition of the vascular endothelial growth factor-A (VEGF-A) to prevent choroidal neovascularization in vasoproliferative retinal diseases, such as AMD. The dual-acting vector showed enhanced therapeutic efficacy with respect to the delivery of a single antiangiogenic factor (Askou et al., 2019).

Despite the known role of VEGF in neovascularization (Folk and Stone, 2010), it has been shown that VEGF treatment reduces apoptosis in models of glaucoma whereas anti-VEGF therapies exacerbate neuronal cell death (Boia et al., 2020). Engineered zinc finger proteins (ZFPs) that upregulate multiple forms of VEGF in their natural, endogenous, ratios were generated to avoid vascular permeability, edema, and inflammation due to VEGF overexpression. AAV-VEGF-ZFPs significantly increased RGCs survival after optic nerve transection or ophthalmic artery ligation without affecting retinal vasculature (D’Onofrio et al., 2011).

Within IR diseases, mutation-independent strategies acting on mitochondrial dysfunction, oxidative stress and mitochondrial-mediated cell death have been proposed to protect RGCs and restore visual function (Figure 2). Delivery of the caspase inhibitor BIRC4 (also known as XIAP), of the antiapoptotic member of the Bcl-2 protein family Bcl-X_*L*_, and of the mitochondrial superoxide dismutase (SOD2), a ROS-detoxifying enzyme, have been tested in *in vivo* models of RGC degeneration with promising results (McKinnon et al., 2002; Malik et al., 2005; Qi et al., 2007).

Interestingly, Xiong et al. (2015) created several AAV vectors to deliver antioxidant genes to the retina. These vectors encode the main mitochondrial ROS scavenger enzymes SOD2 and catalase, and the master transcription factors *NRF2* and *PGC1a*, which globally regulate antioxidant defense responses (St-Pierre et al., 2006). Moreover, PGC1a also regulates mitochondrial biogenesis and respiration (Wu et al., 1999). Notably, AAV-mediated delivery of *NRF2* was more effective than SOD2 and catalase in the protection of both photoreceptor and RGC degenerative models, while surprisingly, expression of PGC1a accelerated photoreceptor death (Xiong et al., 2015). These results suggested that the pleiotropic effect of *NRF2* may be broadly applicable to many ocular diseases characterized by oxidative damage.

### MicroRNA-Based Approaches

Several microRNAs (miRNAs) have been associated with retinal development and function as well as with disease pathogenesis (Karali and Banfi, 2019). As an example, a dominant mutation in the mature sequence of miR-204 was linked to a form of RP associated with ocular coloboma (Conte et al., 2015). Recently, it was reported that AAV-mediated subretinal administration of miR-204 preserved retinal function in mouse models of inherited retinal diseases by slowing down photoreceptor degeneration and dampening microglia activation (Karali et al., 2020). MiR-204 represents a paradigmatic example of a promising mutation-independent approach due to miRNA capability to simultaneously modulate multiple molecular pathways involved in disease pathogenesis and progression.

MicroRNAs represent attractive therapeutic targets for complex retinal disorders as well. A growing number of miRNAs were found to be essential in normal and pathological retinal angiogenesis and were reported to be dysregulated in AMD (Wang et al., 2012; Berber et al., 2017; Askou et al., 2018). Indeed, modulations of miR-184, miR-21, miR-31, miR-150 or miR-23/27 were highlighted as a potential therapeutic approach for AMD (Shen et al., 2008; Sabatel et al., 2011; Zhou et al., 2011; Murad et al., 2014).

Due to their pleiotropic effect, miRNA-based therapeutic strategies have also been tested in ONs with promising results. The effect of miR-124 in axon growth of RGCs derived from Müller cells was tested both *in vitro* and *in vivo* in a model of HTG (He et al., 2018). Isolated rat Müller cells were dedifferentiated into retinal stem cells, induced to differentiate into RGCs and then transfected with miR-124 or anti-miR-124. Interestingly, miR-124 promoted axon growth of RGCs differentiated from retinal stem cells. In a rat HTG model, the extent of axon growth was the longest in the miR-124 group and the shortest in the anti-miR-124 group indicating that miR-124 has translational potential for gene therapy of glaucoma (He et al., 2018).

Finally, the potential therapeutic effect of miR-181a and b downregulation was tested in different models of LHON (Indrieri et al., 2019). The miR-181 family, composed of four members, is highly expressed in different regions of the CNS, including retina, where they regulate neurotrophic signaling, axon guidance, immunity and mitochondrial-related pathways (Indrieri et al., 2020). Genetic inactivation of miR-181a/b protects RGCs and ameliorates visual function in different *in vivo* LHON models strongly suggesting that these miRNAs may represent effective mutation-independent therapeutic targets for this disease (Indrieri et al., 2019).

The therapeutic potential of miRNAs for retinal diseases is also demonstrated by the promising results obtained using Extracellular Vesicles (EV). EV is the collective term for secreted vesicles (exosomes, microvesicles, and apoptotic bodies). They are considered signaling mediators between cells due to their capability of transporting and delivering proteins, mRNA, miRNA, and lipids. EV are gaining attraction as a candidate treatment for ocular diseases and have already been tested in many retinal damage models, such as glaucoma, retinal ischemia, autoimmune uveitis, and diabetic retinopathy, with promising results (Mead and Tomarev, 2020). Interestingly, it is becoming evident that the miRNA component of EV plays a relevant contribution to the protective action of EV. It was recently demonstrated that neural stem/progenitor cells (NPCs)-derived exosomes administration delayed photoreceptor degeneration and preserved visual function mainly through the action of a set of 17 miRNAs that suppressed microglial activation and inflammation in retinal degeneration rodent models (Bian et al., 2020).

### CRISPR/Cas9 Approaches

Therapeutic genome editing is considered an ideal strategy for permanent correction of genetic defects and, since the advent of CRISPR (clustered regularly interspaced short palindromic repeats)/Cas9-mediated genome editing, these strategies are advancing rapidly (Cox et al., 2015). Therapeutic application of CRISPR/Cas9 has shown promising outcomes in animal models of retinal diseases (Benati et al., 2020). In particular, an AAV-CRISPR/Cas9 approach was recently used, in a mutation-independent manner, to inactivate VEGF-A and vascular endothelial growth factor receptor-2 in RPE cells, thus abrogating angiogenesis (Huang et al., 2017; Kim et al., 2017a,b). In three different mouse models of OR degeneration, an AAV-CRISPR/Cas9 was used to target the Neural retina-specific leucine zipper (*Nrl*) gene, which acts in concert with *Nr2e3* in determining rod fate. Loss of *Nrl* in rods causes them to acquire cone-like features. The preservation of cone function is a major therapeutic goal to preserve daylight and color vision and ameliorate RP patients’ quality of life. CRISPR/Cas9-mediated *Nrl* loss showed an improvement of photoreceptors survival and function (Yu et al., 2017). Moreover, double CRISPR/Cas9-mediated targeted inactivation of *Nrl* and *Nr2e3* led to a much more significant rescue of photoreceptor degeneration and restoration of visual function over either single gene targeting (Zhu et al., 2017).

### Optogenetic Approaches

When OR degeneration is at the end stage of disease progression and all photoreceptors are lost (Figure 1D), patients will have little chance to benefit from the previously described approaches and the only mutation-independent strategy available based on gene-delivery is represented by optogenetic therapy. Compared to other therapeutic approaches available at this disease stage, i.e., retinal prostheses and stem cells (Dalkara et al., 2016), optogenetics has the advantage of using pre-existing retinal neural synapses. This approach relies on the creation of newly generated photosensors by transferring photosensitive genes to the residual retinal circuitry. Critical points are represented by the types of light sensors to be used and the retinal cells to target (i.e., RGCs, bipolar, and amacrine cells). The possibility of expressing photosensitive molecules in RGCs represents a promising strategy to treat the most advanced stages of disease, when the retinal circuitry is heavily compromised. Optogenetics mostly uses the light-sensitive proteins channelrhodopsin (ChRs), halorhodopsin (NpHR), and melanopsin (Busskamp et al., 2012; Dalkara et al., 2016; Klapper et al., 2016). ChRs2 and melanopsin expression in bipolar cells restored visual function in murine RP models (Lagali et al., 2008; Doroudchi et al., 2011; Mace et al., 2015), while NpHR could be especially helpful to reactivate the inhibitory modulation of RGCs through expression in bipolar cells (Zhang et al., 2007). Several pre-clinical trials have been conducted in murine, canine, and simian models. These trials include different type of optogenetic molecules expressed alone or in combination and present different targeted cell population (Simunovic et al., 2019). Currently, two Phase I/II clinical trial of optogenetics for vision restoration are registered and underway (NCT02556736) or in the patient recruiting status (NCT03326336).

## Conclusion

Gene delivery approaches have an enormous application potential in retinal disorders not only for gene-specific therapeutic strategies (see the recent approval of the clinical use of the Luxturna drug) but also, for mutation-independent strategies. It is obvious that the use of gene-specific approaches is always the first choice of treatment for a genetic retinal disease, whenever possible. However, as discussed above, mutation-independent therapies constitute a valid alternative/complementary approach to overcome some of the main obstacles related to gene-specific ones, starting from the high genetic heterogeneity of these conditions. Gene-based approaches are predicted to considerably expand the successful application of mutation-independent therapies, for inherited and multifactorial forms of retinal disorders thanks to several advantages summarized as follows:

a) a more stable and long-term expression of the therapeutic gene/product, with significant advantages in safeguarding patients’ welfare over approaches requiring repeated intraocular injections/administrations;
b) the possibility to better target, down to specific cell types, the administration of therapeutic products thus minimizing undesired effects, which is particularly relevant when a combined administration of multiple agents is required;
c) the possibility to apply mutation-independent therapies, e.g., those based on genome editing and optogenetics, for which a different delivery route is either less effective or even impossible.

Based on the above considerations, we believe that gene-based mutation-independent strategies will lead in the near future to the expansion of therapeutic avenues for retinal diseases.

## Author Contributions

SC and SB conceived the manuscript. SC and AI wrote the first draft of the manuscript. SB and BF edited the manuscript. All authors contributed to the article and approved the submitted version.

## Conflict of Interest

The authors declare that the research was conducted in the absence of any commercial or financial relationships that could be construed as a potential conflict of interest.

## Footnotes

1 https://sph.uth.edu/retnet/disease.htm
